# Pathologic features and clinical course of a non-functioning primary pulmonary paraganglioma: A case report

**DOI:** 10.1016/j.amsu.2020.05.027

**Published:** 2020-05-27

**Authors:** Angélica Tobón, Mauricio Velásquez, Bladimir Pérez, Valeria Zúñiga, Luz F. Sua, Liliana Fernández-Trujillo

**Affiliations:** a Faculty of Health Sciences, Department of Internal Medicine, Universidad Icesi, Cali, Colombia; b Department of Surgery, Thoracic Surgery Service, Fundación Valle del Lili, Cali, Colombia; c Department of Pathology and Laboratory Medicine, Fundación Valle del Lili, Cali, Colombia; d Clinical Research Center, Fundación Valle del Lili, Cali, Colombia; e Department of Internal Medicine, Pulmonology Service, Interventional Pulmonology, Fundación Valle del Lili, Cali, Colombia

**Keywords:** Paraganglioma, Neuroendocrine tumor, Thoracic surgery video-assisted, Lung cancer, Case report

## Abstract

**Introduction:**

Paragangliomas (PGGL) are rare neuroendocrine tumors arising from non-epithelial extra-adrenal chromaffin cells. They have been described in different sites: abdomen, pelvis, head, neck and thorax. Incidence is very low, occurring in less than 2–8/million per year. PGGL's of the lung are extremely rare, they have a slow growth and present as painless lesions. Biopsy is the method of choice for diagnosis and prognosis.

**Presentation of case:**

This is a 70-year-old woman with chronic cough, with a CT-scan showing a 3.3-cm mass in the left lower lobe. After video-assisted thoracic surgery, histologic findings confirmed a non-functioning pulmonary paraganglioma. We present the clinical, radiological, pathological findings and clinical course.

**Discussion:**

Primary pulmonary PGGL's are extremely rare neuroendocrine tumors with low-grade malignancy, difficult to distinguish from other pulmonary tumors relying only on imaging techniques. In this case, PGGL presented as an incidentaloma during the evaluation of chronic cough. After histological diagnosis, genetic testing are ideally performed to identify somatic or germline mutations that may condition a higher risk of malignancy and metastasis.

**Conclusion:**

PGGL's must be considered when other diagnoses are unlikely due to immunohistochemistry findings. Larger studies in this field are needed to determine the risk factors for its development and to determine which populations have the greatest potential for malignant transformation

## Highlights

• Paragangliomas are rare neuroendocrine tumors derived from non-epithelial extra-adrenal chromaffin cells.
• Paragangliomas from the lung are rare, they have a slow growth and present as non-painful lesions.
• Complete surgical resection is the diagnostic method of choice for solitary primary pulmonary paragangliomas.
• Genetic testing is important to identify somatic or germline mutations.

## 1 Introduction

Tumors originating from chromaffin cells are located in 90% of cases in the adrenal gland and called pheochromocytomas; the remaining are of extra-adrenal origin and are called paragangliomas (PGGL), which are rare neuroendocrine tumors that arise from non-epithelial extra-adrenal chromaffin cells. PGGL have been described in different sites such as abdomen, pelvis, head, neck and thorax [1]. They were previously considered glomus tumors from the carotid solely [2], but more recently according to the WHO-2017 classification (fourth edition), these tumors are divided into two groups: tumors from the first group originate from the parasympathetic system and are located in the head, neck and less frequently thorax and pelvis; they are typically non-functioning. Tumors from the second group originate from the sympathetic system, 85% are located below the diaphragm, occasionally in the thorax and heart and are more likely to be functional. PGGL are very rare, occurring in less than 2–8/million a year [3]. Lung paragangliomas are much more infrequent, thus, very few cases have been described, and diagnosis is often incidental [2,4,5], since they have a slow growth and present as non-painful, non-malignant lesions, with a doubling time of approximately 42 years [6].

As described above, PGGL may be non-functioning or have neuroendocrine activity, secreting catecholamines, similarly to pheochromocytomas, originating paroxysmal symptoms such as palpitations, diaphoresis or headaches. Case reports of invasive behavior have been described, where metastasis to adjacent lymph nodes are found [7], specifically on mediastinal lymph nodes [4]. Radiological differentiation is difficult when compared with other types of benign, malignant or infectious pulmonary lesions (inflammatory pseudo tumor or tuberculomas) [8]. They usually have high tumor vascularization for they present chest-CT enhancement [9]. Therefore, excision biopsy is the best method to achieve a correct therapeutic approach and prognostic evaluation [10]. Non-functioning PGGL must be distinguished from tumors such as carcinoids, and this is where immunohistochemistry is particularly important for a correct diagnosis and treatment. This work has been reported in line with the SCARE criteria [11].

## 2 Presentation of Case

This is a 70-year-old non-smoker woman, who worked as a lawyer. She was admitted to our hospital with one year of chronic cough, mild hyaline expectoration, usually in the morning. She had no rhinorrhea, postnasal drip, gastroesophageal reflux, dyspnea or wheezing. She had a history of hypertension, anxiety disorder and dyslipidemia, and was on amlodipine/valsartan, hydrochlorothiazide, alprazolam, bupropion and atorvastatin. As of family history, her father had laryngeal cancer and died from diabetes complications; and her mother had lung and breast cancer at 68. Physical examination was normal as well as blood count and other laboratory tests (Table 1).

**Table 1:** Laboratory results.

	Result	Reference range
Leukocyte count	4860/μl	4230–9070
Neutrophils	2620/μl	1780–5380
Lymphocytes	1820/μl	1320–3570
Monocytes	6600/μl	30–820
Eosinophils	60/μl	40–540
Basophils	20/μl	10–80
Hemoglobin	15 gr/dL	13.7–17.5
Hematocrit	46.4%	40.1–51
Platelet count	225.000/μl	163.000–337.000
Serum creatinine	0.66 mg/dL	0.67–1.17

**Fig. 1:**
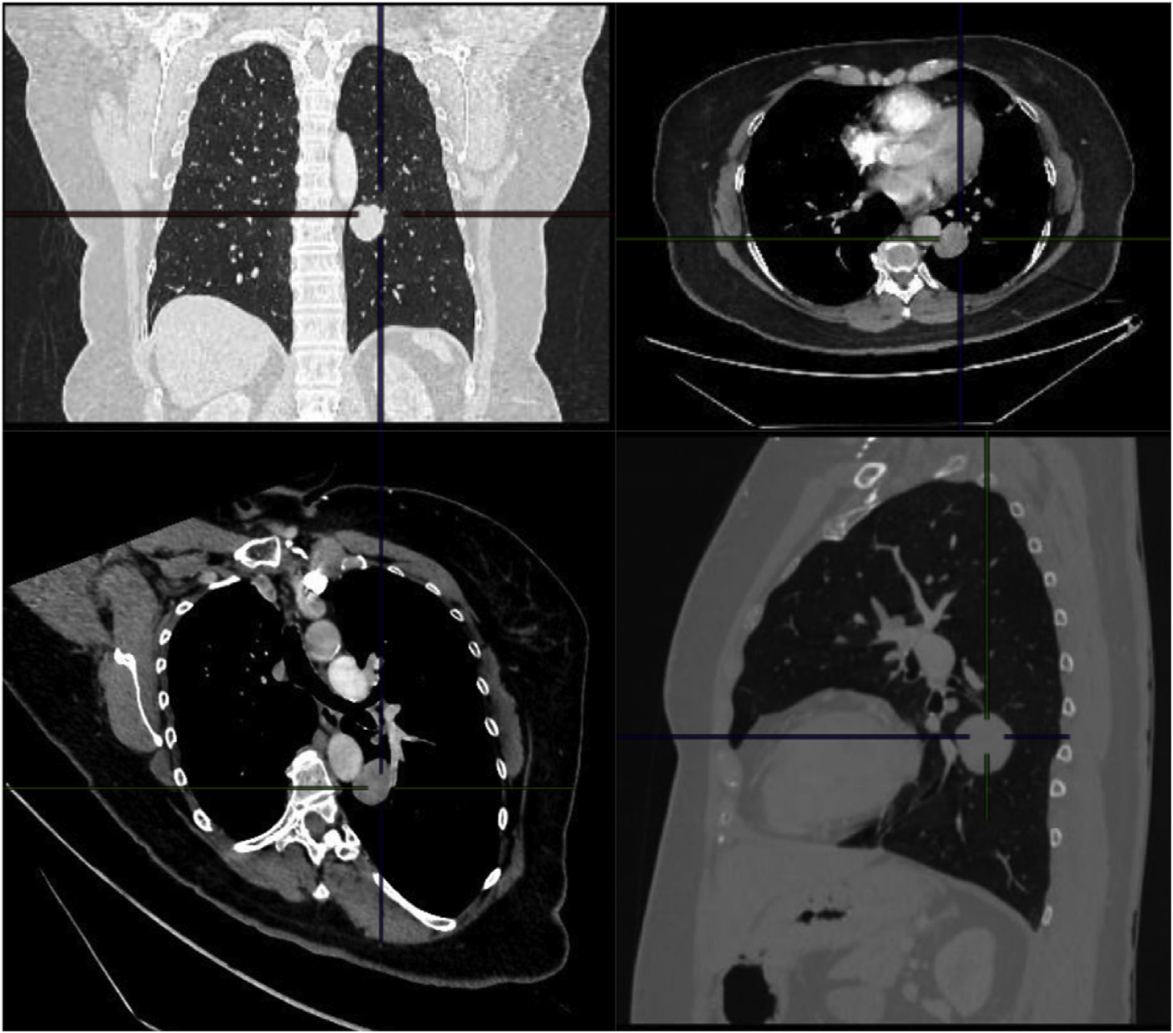
Rounded solid mass of well-defined contours in the apical segment of the lower left lobe (33 × 32 mm), with heterogeneous enhancement. The anterior margin of the lesion is in close contact with the most posterior division of the lower lobar bronchus. In the lateral basal segment of the left lower lobe, a 7.3 mm nodule is observed.

Initially, she was put on antihistaminic medication for two months without improvement. Several complementary tests were performed including a thoracic X-ray that showed a rounded lesion in the left lower lobe. CT scan showed a solid rounded mass with well-defined borders in the apical segment of the left lower lobe (3.3 × 3.2 cm), with heterogeneous enhancement and a 7.3 mm nodule on the left lower lobe lateral segment. The rest of the parenchyma seemed normal, with some mucus plugs in segmental bronchi without dilation (Fig. 1). Abdominal and pelvic imaging was normal, and she was scheduled for surgical resection via video-thoracoscopy with frozen section.

Post-surgical recover had no complications. Since the patient's medical history did not report autonomic symptoms compatible with functioning paraganglioma, such as flushing, palpitations, uncontrolled hypertension, syncope or unexplained diaphoresis; along with the benign appearance in the pathological assessment, we decided that complete resection of the tumor as the definitive treatment and no other therapeutic interventions were considered. The patient has had follow-up outpatient consultations with a satisfactory progress and a complete recovery without complications or additional symptoms.

In histological sections, a solid neoplasm of epithelioid morphology was observed, organized in an organoid pattern with arrangement of small nests delimited by thin fibrovascular septa (sustentacular cells). The tumor was composed of small cells, with a small cytoplasm, central nucleus and granular salt-and-pepper chromatin, without a visible nucleolus. No mitosis was identified in low magnification microscopy; neither dotted nor geographic necrosis was observed.

**Fig. 2:**
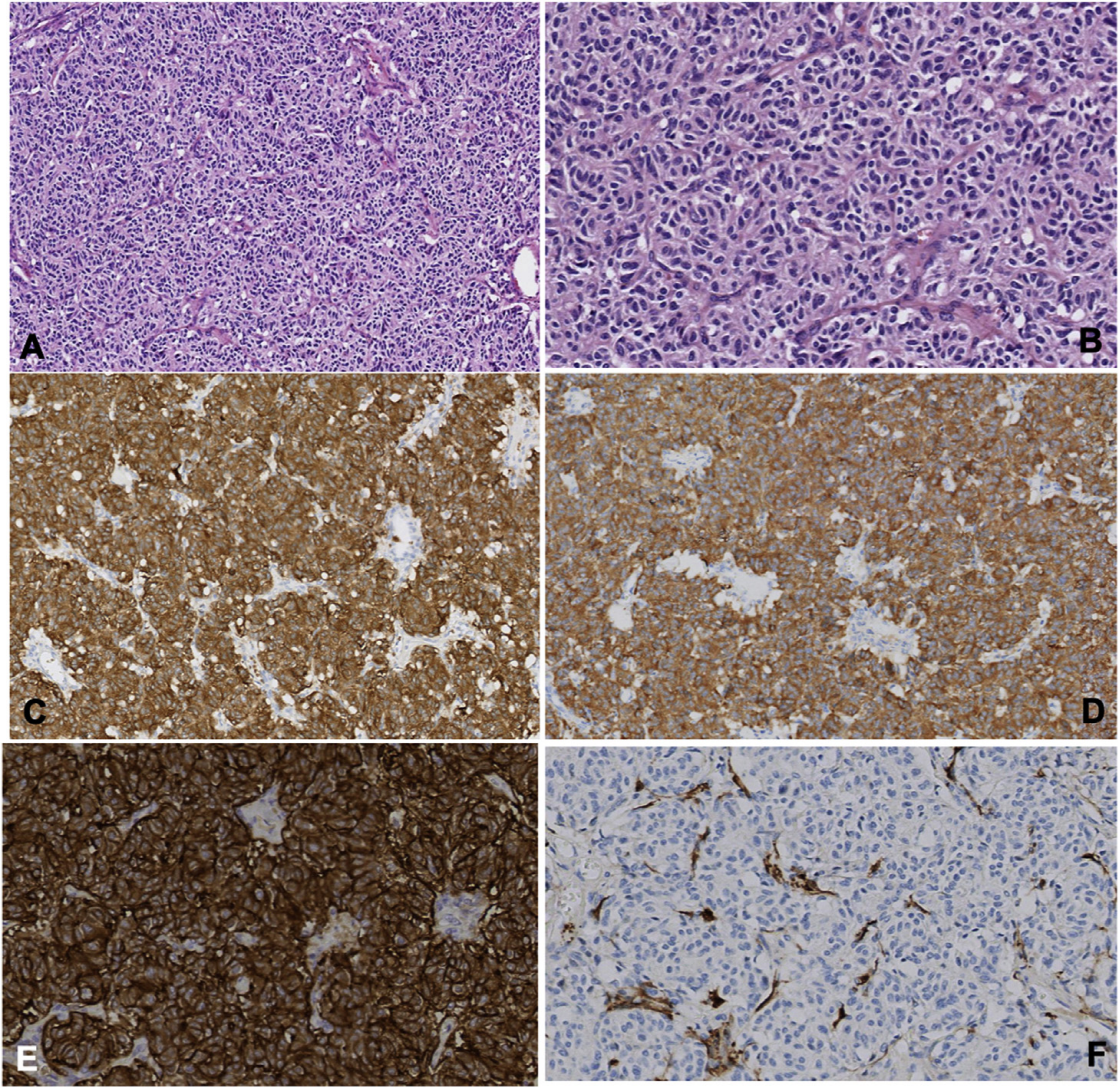
Primary pulmonary paraganglioma. A. H&E 10X, B. H&E 20X. A neoplastic lesion arranged in solid nests. These are surrounded by elongated cells, with eosinophilic cytoplasm and spindle-shaped nuclei (sustentacular cells). Neoplastic cells have neuroendocrine nuclear characteristics, without cytological atypia or mitosis. C. Chromoganin, 10X. Neoplastic cells with neuroendocrine pattern present intense cytoplasmic and global positivity for Chromogranin (neuroendocrine differentiation marker). D. Synaptophysin, 10X. Neoplastic cells with neuroendocrine pattern present, intense cytoplasmic and global positivity for Synaptophysin (neuroendocrine differentiation marker). E. CD56 20X. Neoplastic cells with neuroendocrine pattern with membrane positivity for CD56 (neuroendocrine differentiation marker). F. S-100 protein, 20X. The cells surrounding the tumor nests (sustentacular cells) have cytoplasmic positivity for S-100 protein.

Immunohistochemical staining showed intense and global positivity for CD56 in epithelioid cells. Chromogranin, Synaptophysin and focal positivity for GATA-3. Histological evaluation reported S-100 positive in sustentacular cells. CKAE1/AE3, CK7, CK20, TTF-1, BCL-2, CD34 and STAT-6 were all negative. Ki67 proliferation index was 2%. No mitosis was observed using pHH3 marker (Fig. 2). The final diagnosis was of intrapulmonary benign paraganglioma with clear resection margins.

## 3 Discussion

In this case report, we with highlight the presentation of a rare cancer as primary paraganglioma of the lung found as an incidentaloma during the diagnostic approach of chronic cough, with video thoracoscopy intervention which is now preferred over other techniques [8]; and after pathologic assessment, complete resection was performed as definitive treatment. In this case, video-thoracoscopy was used for its capacity to achieve complete tumor resection with a less invasive procedure compared to conventional thoracotomy. Nevertheless, fine-needle aspiration (FNA) has also been described as a minimally invasive tool to evaluate PGGL, being successful in case series [9]. Cytologic diagnosis, however, can be challenging because of the rarity of these neoplasms and overlapping morphologic features. Therefore, on-site evaluation is very important to ensure adequacy of cells for cell block preparations when FNA is used.

Most primary pulmonary paragangliomas are non-functioning and usually do not show clinical symptoms. Some reported symptoms are cough and chest pain, but the population at risk is unknown [10]. Such is the case of our patient, who presented very mild and nonspecific symptoms. There are no serum markers or radiological characteristics guidelines for the diagnosis of pulmonary non-functioning paraganglioma, and unfortunately, it can be difficult to identify specific signs for this disease. Specially, paragangliomas that emerge in unusual anatomic locations can be a major source of confusion and diagnostic error.

Although most PGGL are benign, 25% may present malignant characteristics and some of them can be misdiagnosed as lymphomas, carcinomas or neuroendocrine tumors metastatic to lymph nodes [12,13]. In this case, no evidence of metastasis to other organs or lymph nodes was found. No mitosis was detected with the pHH3 marker, neither necrosis, nor high Ki-67 proliferation index. Therefore this tumor was considered benign. It is important to highlight that differential diagnosis includes lung cancer, metastatic lung tumors, carcinoid tumor, hamartomas and other inflammatory lesions such as inflammatory pseudo tumor or tuberculomas, and solitary fibrous lung tumors.

During histological and immunohistochemistry (IHC) assessment, multiple markers allow differentiation between different types of neoplasms. BLC2 STAT-6 and CD34 are positive in solitary fibrous lung tumor. CK AE1/AE3, CK7 and TTF-1 are positive in carcinoma. CK AE1/AE3 and CK7 are positive in carcinoid tumors. PGGL and pheocromocytomas are positive for neuroendocrine markers including synaptophysin and chromogranin A, while sustentacular cells are positive for S100, although these cells are frequently absent. Inhibin, melan A, and calretinin are typically negative in PGGL and show diffuse positive staining in adrenal cortical tumors. Similar to PGGL, neuroendocrine tumor shows positive staining for chromogranin and synaptophysin; however, PGGL is usually negative or only shows focal or weak expression for cytokeratins such as CAM5.2 [13].

IHC is useful to confirm PGGL diagnosis, but it is not sensitive or specific to determine whether it is functional or not. Laboratory studies are required to demonstrate an increase in serum metanephrines and other metabolites, along with clinical presentation that arises suspicion of a functional PGGL. If sympathetic symptoms are not present, PGGL diagnosis can be quite a challenge, especially when it presents in unusual locations such as the lung or liver [14].

PPGLs are recognized to have the highest degree of heritability of any endocrine tumor type. The pan-molecular characterization of the Cancer Genome Atlas (TCGA) provides a molecular taxonomy for PGGL. Different molecular subgroups and driver mutations end up in different clusters: 1) pseudohypoxia group is divided in two subgroups: tricarboxylic acid (TCA) cycle–related, containing germline mutations in SDHA, SDHB, SDHC, and SDHD as well as SDHAF2 (SDHx), and FH; and VHL/EPAS1-related, with somatic and germline mutations. 2) Wnt signaling group includes somatic mutations in CSDE1 and somatic gene fusions of MAML3; and 3) kinase signaling group includes germline or somatic mutations in RET, NF1, TMEM127, MAX, and HRAS [15].

In this case, assessment for genetic mutations was not performed due to non-availability of the test in our hospital, for it is performed outside the country. Nonetheless, since hereditary PGGL is estimated to be as high as 40% and approximately 16% have SDHx or FH mutations [[16], [17], [18], [19]] and certain mutations are associated to a higher malignant risk, it is very important to evaluate for genetic predisposing syndromes and driver mutations in PGGL patients. Similarly, in this case, since the possibility of a pheochromocytoma or a functioning PGGL was not suspected due to the unspecific nature of the symptoms (chronic cough alone), plasma metanephrines were not measured pre-operatively and PGGL diagnosis was purely incidental. Additionally, the patient's arterial hypertension and anxiety disorder did not resolve after resection of the tumor. She was followed at six months with chest-CT scan and no evidence of recurrence was found.

## 4 Conclusions

Primary pulmonary paragangliomas are rare non-functioning neuroendocrine tumors, most of them benign, difficult to distinguish radiologically from other lesions, but must be considered when revising a non-typical histologic report, where other diagnoses are not likely due to immunohistochemistry findings. Larger studies in this field are needed to determine the risk factors for its development and to determine which populations have the greatest potential for malignant transformation, besides of the already known mutations associated with metastatic tumors.

## Ethics approval and consent to participate

This report was prepared in accordance with the ethical standards of the institutional ethics committee and with the 1964 Helsinki Declaration. We have approval letter of Ethics Committee in biomedical research IRB/EC No. 268–2019 of the Fundación Valle del Lili to publish this manuscript.

## Funding

No funding sources were used.

## Authors' contributions

All authors have significantly contributed to the paper: AT: Conception and design, literature review, manuscript writing and correction, final approval of manuscript. MV: Conception and design, literature review, final approval of manuscript. BP: Conception and design, literature review, final approval of manuscript. LFS: Conception and design, literature review, final approval of manuscript. LFT: Conception and design, literature review, manuscript writing and correction, final approval of manuscript.

## Trial registry number

Name of the registry: Does not apply

Unique Identifying number or registration ID:

Hyperlink to your specific registration (must be publicly accessible and will be checked):

## Guarantor

Liliana Fernández-Trujillo., MD.

Department of Internal Medicine

Pulmonology Service, Interventional Pulmonology

Fundación Valle del Lili, Cra 98 # 18–49,Tower 6, 4th Floor

Cali, Colombia. Zip code 7600032. Phone (+57) 3155006300

liliana.fernandez@fvl.org.co, lilianafernandeztrujillo@gmail.com

## Consent for publication

Written informed consent was obtained from the patient for publication of this case report and any accompanying images. A copy of the written consent is available for review by the Editor-in-Chief of this journal.

## Availability of data and materials

All data and material are available for sharing if needed.

## Provenance and peer review

Not commissioned, externally peer reviewed.

## Declaration of competing interest

The authors declare that they have no competing interests. This manuscript has not been published and is not under consideration for publication elsewhere. Additionally, all of the authors have approved the contents of this paper and have agreed to the journal's submission policies.

## List of abbreviations

WHO: World Health Organization
CD56: Archetypal phenotypic marker of natural killer
GATA-3: Transcription factor encoded in humans by the GATA3 gene
CK AE1/AE3: Monoclonal Mouse Anti-Human Cytokeratin Clones AE1/AE3
CK 7: Cytokeratin 7
CK 20: Cytokeratin 20
TTF-1: Thyroid Transcription Factor-1
BCL-2: B-cell lymphoma 2 protein
CD34: Cluster of differentiation 34
STAT-6: Signal transducer and activator of transcription 6
KI 67: The proliferation marker
PGGL: Paraganglioma
PHH3: Phosphohistone H3
FNA: Fine-needle aspiration
